# One of the Successes of the War

**Published:** 1919-03-22

**Authors:** 


### THE SANITARY ORGANISATION OF ARMIES IN THE FIELD.

### One of the Successes of the War.

"During our wars of the last thirty years forty men have become hospital patients, and five have died as the result of disease for every one killed by the enemy" (Lelean). It was quite evident, therefore, that sanitation, or the practical application of the laws of hygiene, which aim at the maintenance of health, would be a very important factor in the present war, and so the event has proved. Fortunately, those who were at the head of the Army Medical Service recognised the need for a definite sanitary organisation overseas at an early date, and their foresight has been amply justified.

### The Sanitary Section.

One of the greatest successes of the war has been in the Sanitary Section, which forms the unit of the present sanitary organisation. Before the war there existed two Territorial formations, the 1st and the 2nd London Sanitary Companies; these provided the nucleus for the majority of the sections which were subsequently formed. The officers of these companies were of two classes. There were some who were doctors, most of them employed in the Public Health Service as medical officers of health, as school doctors, or tuberculosis officers. But there were also a large number of engineers, architects, surveyors, and analytical chemists, with special knowledge and experience in sanitary matters, who also held commissions in these companies. The rank and file were trained in sanitary work, as well as receiving the usual training of the R.A.M.C. in first-aid, stretcher drill, etc. Each section, as it was formed, was composed of an officer with the rank of lieutenant or captain and twenty-five non-commissioned officers and men. The equipment of a section included spades, pick-axes, brooms, buckets, watering-pots (for spraying floors), a fitted tool-chest, a water-testing case, a filter for water, two box disinfectors, mason's trowels, sprayers (for disinfectants), a couple of bicycles (increased to four later—officially—though unofficially there were often more than four), and a few odd tools of various kinds. The whole equipment was packed and carried in a 30-cwt. motor lorry, for which two drivers were attached to each section.

### "Army" Units *v.* the Divisional Régime.

During the summer of 1917 the lorry was taken away from each section and was replaced by a Ford box-car and a motor-cycle with a side-car. Originally the officer was provided with a horse. In the beginning it was only intended, as laid down in the War Establishment, to have a sanitary section at each base of the field force, but it was very soon found necessary to extend the sphere of activities right up to the front line. Fresh sections were accordingly sent out from England early in 1915, and, in addition, as new divisions were despatched overseas, they were each provided with a sanitary section. In some cases these were raised from the same district as the division, and not from the London companies. For instance, a Midland division was provided with a section largely drawn from Birmingham. The officer was a health official, the N.C.O.s were sanitary inspectors and instructors in the technical schools of that city.

This was an exceptional case; not many units were composed of such suitable material. At first each sanitary section which was not employed at a base was attached to a division, and moved with the division wherever it went. During the battle of the Somme, however, it was found that the constant movements and reliefs of divisions which took place interfered with the continuity of the work of the sanitary sections. They were accordingly made "army" units, and to each was allotted a part of the army area, irrespective of what troops were actually occupying the area. This change only took place in France; elsewhere the sections continued under the divisional *régime*. In the later stages of the war, when our troops were rapidly advancing, it was found more convenient to revert to the original arrangement. The "area" distribution worked very well, for it enabled a section to get to know its district thoroughly and to equip it completely with every sort of sanitary appliance. On the other hand, when each section was attached to a division, through being known by every unit of the division they were able to get what they wanted done more readily and with less friction than if they had been dealing with strangers. The personal equation, as always, was an important factor in this respect.

### Duties of a Sanitary Section.

Now as to the duties of a sanitary section. Firstly, they are inspectors. It is the duty of the officer and his N.C.O.s to visit all camps and billets in their area and see that all orders as to sanitation are being obeyed. If they find anything wrong it is brought to the notice of the officer commanding the battalion or battery, or other unit as the case may be, and he is asked to have matters put right. But a large part of the time and energy of a sanitary section is occupied with work which is not strictly theirs at all. The provision of sanitary appliances such as washing-benches, incinerators, latrine-seats, etc., is one of the duties of the Royal Engineers. It was very soon found that during fighting the "sappers" were fully occupied with defence works, wiring trenches, and making dug-outs, etc., so that it was impossible for them to find time to make anything like the number of appliances required by a division in the field. And so the sanitary sections took over this work also. Workshops were established at their headquarters, skilled N.C.O.s were put in charge of the carpentry and metal shops, and under their guidance labourers—often unskilled—worked, being provided by the different units of the division. In addition, the work of a Sanitary Section includes the disinfection of billets and the supervision of a variety of other matters such as bathing arrangements, the burial of carcases, the cleansing of verminous clothing, and the disposal of sullage and ablution water.

These Sections are the only organised sanitary units, but each battalion, and its equivalent in other branches of the Service, has a small number of men detailed for sanitary duties, who are trained by the Regimental Medical Officer and under whose supervision they work. There are also "squads" responsible for the sanitation at Railheads. The general principle of sanitation in the Army is that the Commanding Officer is responsible for the sanitation of his unit, not the Medical Officer, whose function is merely advisory, not executive.

"Blue Tabs."

